# Motor‐induced oscillations in choice response performance

**DOI:** 10.1111/psyp.14172

**Published:** 2022-08-30

**Authors:** Ulrich Pomper, Ulrich Ansorge

**Affiliations:** ^1^ Department of Cognition, Emotion, and Methods in Psychology, Faculty of Psychology University of Vienna Vienna Austria; ^2^ Cognitive Science Research Hub University of Vienna Vienna Austria; ^3^ Research Platform Mediatised Lifeworlds University of Vienna Vienna Austria

**Keywords:** alpha rhythm, attention, cyclic, mu, psychophysics, visual

## Abstract

Recently, numerous studies have revealed 4–12 Hz fluctuations of behavioral performance in a multitude of tasks. The majority has utilized stimuli near detection threshold and observed related fluctuations in hit‐rates, attributing these to perceptual or attentional processes. As neural oscillations in the 8–20 Hz range also feature prominently in cortical motor areas, they might cause fluctuations in the ability to induce responses, independent of attentional capabilities. Additionally, different effectors (e.g., the left versus right hand) might be cyclically prioritized in an alternating fashion, similar to the attentional sampling of distinct locations, objects, or memory templates. Here, we investigated these questions via a behavioral dense‐sampling approach. Twenty‐six participants performed a simple visual discrimination task using highly salient stimuli. We varied the interval between each motor response and the subsequent target from 330 to 1040 ms, and analyzed performance as a function of this interval. Our data show significant fluctuations of both RTs and sensitivity between 12.5 and 25 Hz, but no evidence for an alternating prioritization of left‐ versus right‐hand responses. While our results suggest an impact of motor‐related signals on performance oscillations, they might additionally be influenced by perceptual processes earlier in the processing hierarchy. In summary, we demonstrate that behavioral oscillations generalize to situations involving highly salient stimuli, closer to everyday life. Moreover, our work adds to the literature by showing fluctuations at a high speed, which might be a consequence of both low task difficulty and the involvement of sensorimotor rhythms.

## Abstract 2

Numerous studies have revealed 4‐12 Hz fluctuations in performance, using stimuli close to detection threshold. Here we report oscillations in response times and sensitivity at 12‐25 Hz, employing a simple task and highly salient stimuli. We conclude that the phenomenon can be generalized to situations closer to everyday life, while the higher frequency likely reflects both reduced task difficulty and the involvement of sensorimotor neural oscillations.

## 1 INTRODUCTION

Fast and accurate motor responses are crucial for survival and successful task performance in countless situations. While both speed and accuracy are widely studied in psychological and neuroscience experiments, they exhibit considerable within‐subject variability, which is commonly accredited to neural noise during sensory processing, decision‐making, and response generation (Dmochowski & Norcia, 2015; Hanes & Schall, 1996; Luce, 2008; MacDonald et al., 2006; Wood, 1977). More and more, however, research is starting to explain parts of this variability, including its underlying physiological mechanisms (e.g., Benedetto et al., 2021; Bompas et al., 2015; Helfrich et al., 2018; Johnson et al., 2015; Lakatos et al., 2008; Paraskevopoulou et al., 2021; Ribeiro et al., 2016). In the present study, we consider the idea, that particularly variability in reaction times (RTs) might be explained by ongoing rhythmic fluctuations of neural excitability in motor areas and the resulting capacity to initiate manual responses.

Overall, accumulating evidence suggests that human neural processing capacity is subject to cyclic fluctuations over time (Benedetto et al., 2019; Fiebelkorn et al., 2013; Fiebelkorn & Kastner, 2019; Landau & Fries, 2012; Pomper & Ansorge, 2021; VanRullen, 2016). For instance, electrophysiological studies have demonstrated that perception depends on the phase of ongoing low frequency (<14 Hz) oscillatory neural activity prior to the onset of a stimulus (Busch et al., 2009; Davis et al., 2020; Ng et al., 2012; Sokoliuk & VanRullen, 2011; VanRullen & McClelland, 2013; Wyart et al., 2012). As neural oscillations reflect the excitability of the underlying tissue, stimuli occurring at a high excitability phase are more likely to be processed in a fast and accurate manner than stimuli occurring at a low excitability phase (Fries, 2015; Schroeder & Lakatos, 2009).

Recently, these findings were corroborated via purely behavioral studies, in which task performance is shown to fluctuate rhythmically over time (Benedetto et al., 2018; Fiebelkorn et al., 2013; Landau & Fries, 2012; Pomper & Ansorge, 2021; VanRullen et al., 2007; Wang et al., 2020). Such experiments usually employ a so called “dense sampling” approach, in which performance is assessed at many close‐spaced intervals following a salient “reset” event. For example, Landau and Fries (2012) presented their participants with a Posner spatial cuing paradigm, and systematically varied the delay between an uninformative left or right cue and a subsequent left or right target. They observed that target detection performance fluctuated rhythmically at 4–10 Hz as a function of the cue‐to‐target delay interval, suggesting that the salient cue reset ongoing oscillations in neural excitability. Interestingly, this and other studies have observed that during tasks which require the simultaneous monitoring of two locations or objects, the observed fluctuations in performance at each location or object are out of phase with each other, suggesting they are processed in alternation (Fiebelkorn et al., 2013; Landau & Fries, 2012; Re et al., 2019). Beyond attention toward external stimuli, we have recently demonstrated that representations in visual working memory are also subject to ~6 Hz rhythmic fluctuations in fidelity, and that two simultaneously memorized representations seem to be prioritized in alternation (Pomper & Ansorge, 2021).

Overall, this suggests that cyclic variations in information processing capacity may be a general characteristic of the brain, potentially linked to limited processing resources, such as during attentional monitoring or short‐term maintenance of task‐relevant information.

However, little is known regarding whether manual RTs are likewise contingent upon rhythmic excitability fluctuations in motor‐related brain areas, independent of preceding perceptual or attentional processes (cf. Tan et al., 2016). While a number of behavioral dense‐sampling studies have reported rhythmic fluctuations in RTs, virtually all of them employed difficult detection or discrimination tasks with target stimuli close to perceptual threshold (e.g., Benedetto & Morrone, 2017; Diederich et al., 2012; Drewes & VanRullen, 2011; Helfrich et al., 2018; Peters et al., 2021). As such, these experiments were optimized to detect variability at the perceptual and/or attentional rather than at the response level. Consequently, their results are commonly interpreted as reflecting perceptual or attentional fluctuations, leaving it unclear whether and to what degree they are additionally affected by temporal variability in response initiation.

Importantly, the presence and functional relevance of neural oscillations in the range of 8–13 Hz (alpha/mu rhythm) and 14–30 Hz (beta rhythm) in cortical motor areas during movement preparation and execution is well established (Desideri et al., 2019; Hussain et al., 2019; Kilavik et al., 2013; McFarland et al., 2000; Pogosyan et al., 2009; Stolk et al., 2019; Tzagarakis et al., 2010). Assuming that these oscillations also constitute patterns of waxing and waning neural excitability, it seems reasonable that the speed of overt behavioral responses is partly contingent on the phase of these ongoing fluctuations.

While evidence for such a mechanism from electrophysiological studies is mixed and not unambiguously attributable to the motor system (e.g., Bompas et al., 2015; Diederich et al., 2014; Drewes & VanRullen, 2011), recent strong support comes from animal studies (e.g., Lacey et al., 2014) and experiments in humans directly probing ongoing changes in the excitability of cortical motor areas via transcranial magnetic stimulation (TMS) (Berger et al., 2014; Keil et al., 2014; Khademi et al., 2018). For instance, Khademi et al. (2018) found that the amplitude of TMS‐evoked motor potentials depends on the phase of 14–24 Hz oscillations in motor areas, prior to TMS pulse application.

Taken together, previous research supports the assumption that the commonly observed intraindividual variability in RTs can be partly explained by cyclic fluctuations of excitability in cortical motor areas. Additionally, an intriguing possibility is that in situations, in which the brain has to simultaneously prepare for a speeded motor response with just one of two possible effectors (e.g., the left or right hand), the capacity to do so might cyclically alternate between them. Such a mechanism would be in line with the alternating attentional sampling of spatial locations, objects or working memory items discussed above (Fiebelkorn et al., 2013; Landau & Fries, 2012; Pomper & Ansorge, 2021) and reflect an efficient response preparation strategy in the light of limited resources. In the present study, we set out to further investigate these possibilities by employing a simple and straightforward speeded RT task along with a highly salient, above threshold target stimulus, aiming to reduce the potential impact of perceptual or attention related fluctuations. Specifically, we were interested in two research questions: (1) Can we observe systematic fluctuations in RTs (predominantly related to motor processes) independent of fluctuations in sensitivity (i.e., related to perceptual or attentional processes)? (2) If so, are RT fluctuations for concurrently expected left‐ and right‐hand responses in counterphase, indicative of a cyclic alternating preparation of two potentially task relevant effectors?

Our participants were asked to provide speeded responses regarding the direction of large, high‐contrast arrows. By densely varying the interval between each motor response and the presentation of the next target stimulus, we were able to estimate ongoing fluctuations of both RTs and sensitivity over time, independently for the two hands.

We observed motor‐induced rhythmic fluctuations of both RTs and sensitivity at 12–20 Hz and 14–25 Hz, respectively, but no evidence for an alternate prioritization of left‐ and right‐hand responses. Thus, our results demonstrate an impact of motor signals on behavioral oscillations, but might be additionally shaped by perceptual or attentional mechanisms earlier in the target processing hierarchy.

## 2 MATERIALS AND METHOD

### 2.1 Participants

Twenty‐six participants (including the first author) took part in the experiment, either in exchange for course credits or monetary compensation. Our sample size was based on previous studies on attentional sampling, which commonly incorporated between 15 and 25 participants (e.g., Fiebelkorn et al., 2013; Ho et al., 2017; Landau & Fries, 2012; Pomper & Ansorge, 2021).

All participants (five males; *M*_age_ = 21.9 years, *SD*_age_ = 4.1) had normal or corrected to normal vision and were naive to the purpose of the experiment. All gave written informed consent, and the study was conducted in accordance with the standards of the Declaration of Helsinki. We further followed the Austrian Universities Act, 2002 (UG2002, Article 30 § 1), which states that only medical universities or studies conducting applied medical research are required to obtain an additional approval by an ethics committee. Thus, no additional ethical approval was required for our study.

### 2.2 Experimental setup and task

Stimuli were displayed on a 24.5‐inch LCD monitor with a resolution of 1280 by 1024 pixels and a refresh rate of 100 Hz. The experiment was run via OpenSesame (Version 3.2.8; Mathôt et al., 2012) on a PC running Windows 7. Participants sat inside a dimly lit room 57 cm away from the screen, with their heads supported by a chin‐ and forehead rest, and wearing earmuffs to cover potential clicking sounds elicited by their button presses. Visual stimuli were presented against a gray background (luminance: 11.6 cd/m^2^) and a central fixation dot (0.19° diameter) was displayed throughout the experiment.

**FIGURE 1:**
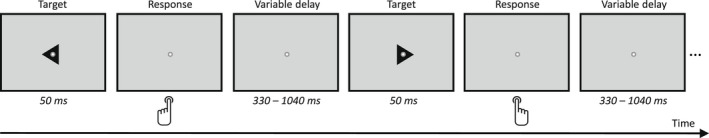
Experimental design. Participants were presented with a sequence of visual triangles, pointing either to the left or right. The task was to provide a speeded response with the index finger of hand toward which the triangle was pointing (left or right). Each button‐press then triggered a variable delay interval ranging from 330–1040 ms, after which the next triangle was presented

At the beginning of each trial (Figure 1), a black equilateral triangle (side‐length: 1.48° diameter; luminance: 2.2 cd/m^2^), was presented centrally for 50 ms, pointing either to the left or to the right. The task was to provide a speeded response with the index finger of the hand (left or right), toward which the triangle was pointing. Responses were given via the “y” and “:” keys of a conventional “qwert” keyboard and were deemed valid when given within 1 s following the offset of the triangle. Each motor response was followed by a variable inter‐trial interval (ITI) of 330 to 1040 ms duration (in steps of 10 ms), after which the next target triangle was presented. We tested if the motor response acts as a resetting event to ongoing rhythmic fluctuations in neural excitability, so that the variable ITI would allow us to estimate periodical fluctuations in performance with high temporal resolution.

Each participant completed two experimental sessions on separate days, split into 10 to 12 blocks with sufficient breaks in between. An average of 2994.2 trials (*SD* = 135.2, range = 2664 to 3096) was presented per participant, resulting in an average of 41.6 trials per time window (*SD* = 1.9, range = 37 to 43). Note that unequal trial numbers were due to technical difficulties during the experiment (*N* = 4), as well as the decision to increase trial numbers from 2904 to 3106 (*N* = 15), as one experimental session turned out to last less than the scheduled 60 min.

An equal number of trials requiring left‐ and right‐handed responses was presented. The order of trials was randomized, with the restriction that no more than four trials with the same target direction were presented in succession. The latter led to an overall higher number of trials requiring a different (*incongruent* condition) rather than the same (*congruent* condition) response than the previous trial (1768 versus 1462, respectively, on average). Prior to the main experiment, each participant completed a short training block. Participants were instructed to respond as fast as possible and keep errors at a minimum.

### 2.3 Data analysis

All analyses were performed using Matlab (2018, Mathworks inc., Natick MA) and the CircStat toolbox (Berens, 2009). First, for each participant, we removed outlier trials with response times (RTs) deviating more than 2.5 *SD*s from the mean (*M* = 3.1% *SD* = 0.67%). Sensitivity (*d’*) was computed as the difference between the *z*‐transformed hit‐rate minus the *z*‐transformed false alarm rate. For descriptive purposes, we then calculated mean RTs (for correct trials only) and *d’* pooled across all trials, as well as separately for the congruent and incongruent condition. The latter two were further compared using paired *t*‐tests.

To estimate rhythmic fluctuations in performance over time, we investigated the time course of RTs and *d’* as a function of ITI, starting from the button‐press to the previous target stimulus. Thus, for each participant, we first sorted all trials according to the duration of the ITI. Using a moving‐window approach with a 10 ms step‐size, we then computed the average RT and *d’* within bins of five consecutive delay‐period intervals (i.e., within 50 ms) (Fiebelkorn et al., 2013; Pomper & Ansorge, 2021). This was done for data pooled across all trials, as well as separately across trials from the congruent and incongruent condition. As an example, we first computed the performance for trials with an ITI between 330 to 380 ms. Then, we shifted the time window by 10 ms and computed performance for trials with an ITI between 360 to 390 ms. This procedure was performed throughout the entire duration of possible ITIs. In order to maximize the length of the timeseries and the resulting frequency resolution of the spectral decomposition, we included the final four ITIs from 1000 to 1040 ms, which due to the nature of the moving‐window procedure, comprised fewer datapoints than the preceding intervals. While all other ITIs contained a mean of 204.4 trials per participant, the last four contained a mean of 182.0, 160.2, 133.3, and 111.0 trials, respectively. Next, we de‐trended and normalized the resulting single‐subject RT and *d’* time courses by subtracting the second order polynomial fit and performed Fast Fourier Transform to estimate their spectral composition. This resulted in both power and phase values of 19 frequency bins from 0 to 25 Hz, separately for each participant.

To statistically test for the presence of rhythmic temporal fluctuations in the performance time courses (i.e., peaks in their power spectra), we applied a nonparametric resampling procedure. We randomly reshuffled RTs as well as hits and misses across all ITIs within each participant. Then we performed the same analysis steps on the reshuffled data as described above for the empirical data and repeated this procedure 1000 times. This created a distribution of 1000 power values for each frequency bin, from which we determined the statistical thresholds at *p* = .05, and applied FDR correction for all frequency bins (Benjamini & Hochberg, 1995). Thus, only spectral peaks in the empirical data exceeding 95% of the surrogate data peaks were considered significant. As an additional, independent assessment of the spectral properties of our data, we also performed a logistic regression analysis, by fitting a series of sines and cosines to each participant's time series data (e.g., Tomassini et al., 2017). The details and outcomes of this procedure, which corroborate our main analysis, are reported in the Supplementary Materials (Figure S1).

Finally, we were interested in a potential phase consistency of performance fluctuations across participants. As each datapoint in the time‐series indicates a target onset, the phase angle of the time‐series reflects the performance at target onset. For any observed significant spectral peak, we computed a Rayleigh test for non‐uniformity of circular data (Berens, 2009), which assesses whether the phase angles from the individual participants are distributed uniformly around the unit circle or not. This test was again performed on both the empirical and the surrogate data, and statistical significance was assumed if the *p* value from the empirical data exceeded 95% of *p* values in the surrogate data.

## 3 RESULTS

**FIGURE 2:**
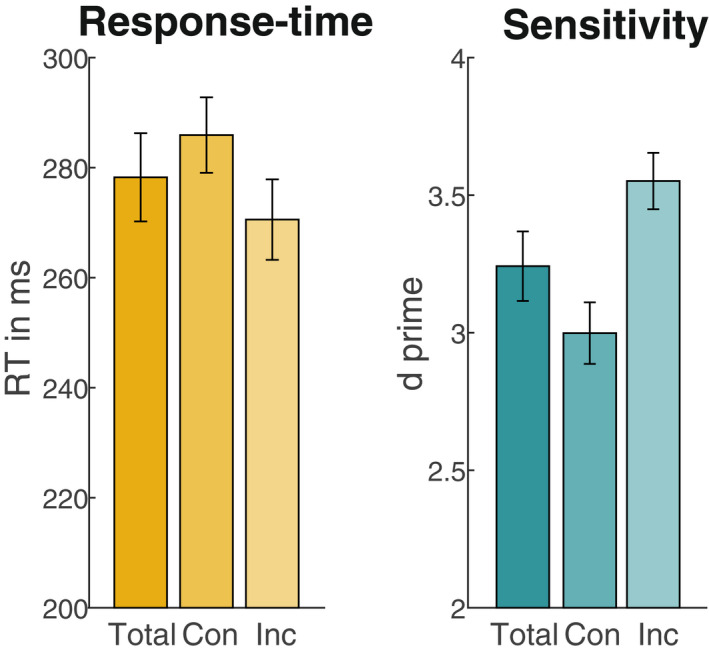
Overall behavioral results. Left: Response times (RTs) pooled across conditions, as well as separately for trials with response sides congruent (con) or incongruent (Inc) with the previous trial. Right: Same as left, for sensitivity (*d prime*). Con: Congruent; Inc: Incongruent

Participants performed well overall, with an average RT of 278 ms and *d’* of 3.24 (Figure 2). Responses were both significantly faster (*t*_1,25_ = −4.98, *p* < .001) and had a higher sensitivity (*t*_1,25_ = 4.62, *p* < .001) for incongruent‐compared to congruent trials.

**FIGURE 3:**
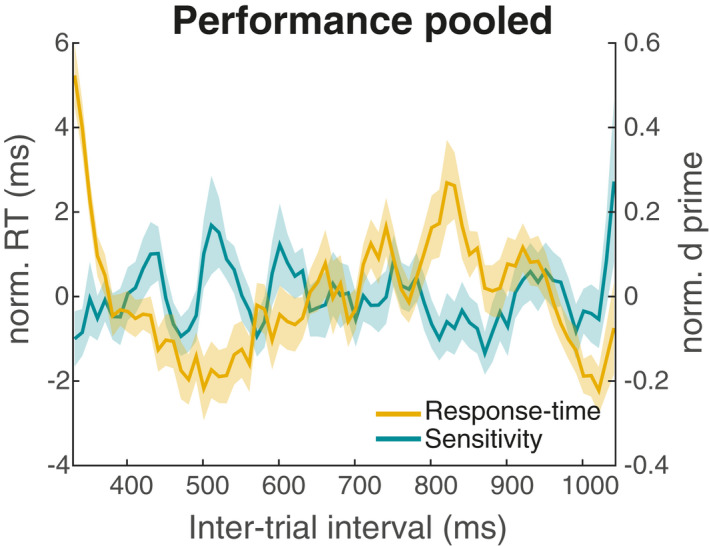
Grand‐average performance time course. Average response times (RTs, yellow trace) and sensitivity (green trace) as a function of the variable inter‐trial interval (ITI), pooled across all trials. Shaded areas indicate *SEM*. Norm.: Normalized

**FIGURE 4:**
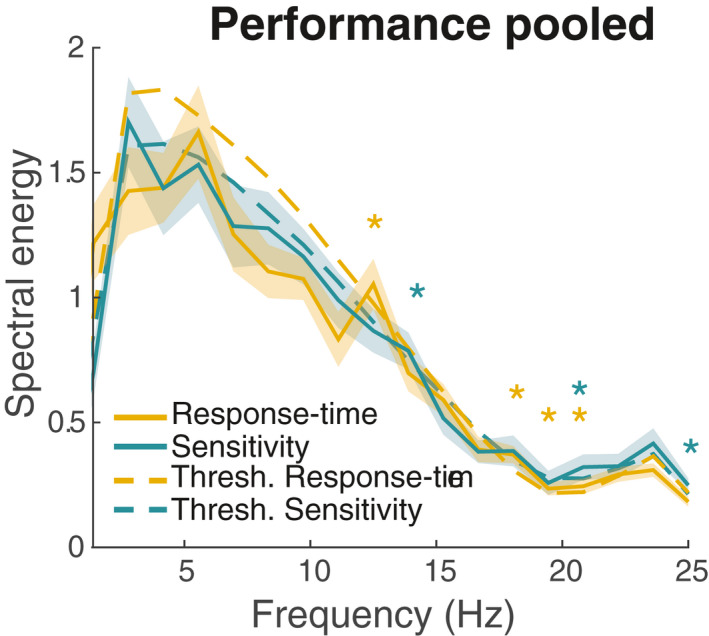
Grand average spectral representation of individual performance time courses. Yellow traces indicate the power spectrum for fluctuations in response times, green traces for fluctuations in sensitivity. Solid lines represent the empirical data, dashed lines the threshold (thresh.) determined from resampled surrogate data. Significant deviations (corrected for multiple comparisons) are marked by an asterisk. Shaded areas indicate *SEM*

Figure 3 illustrates the grand‐average time‐course (pooled across all trials) of both RTs and *d’* (power spectra for each individual participant are shown in Figure S2). Fluctuations depending on the ITI are evident in both measures. Looking at the spectral representation (Figure 4), we observed significant peaks for RTs at 12.5 Hz, 18.1 Hz, 19.4 Hz, and 20.8 Hz (*p* = .030, *p* < .001, *p* = .030, and *p* = .019, resp.). For sensitivity, the analysis yielded significant peaks at 13.9 Hz (*p* = .048), 20.8 Hz (*p* = .019), 23.6 Hz (*p* = .025), and 25 Hz (*p* < .001). Thus, for both RTs and sensitivity grand‐average time courses, we observed significant spectral peaks at around 12–14 Hz (i.e., the alpha‐band range) and around 18–25 Hz (i.e., the beta‐band range).

**FIGURE 5:**
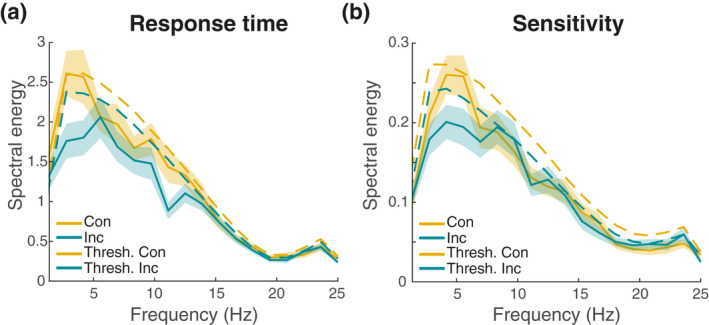
Grand average spectral representation of performance separately for congruent and incongruent trials. (a) Spectra for response times: Yellow traces indicate the power spectrum for fluctuations in congruent trials, green traces for fluctuations in incongruent trials. Solid lines represent the empirical data, dashed lines the resampled surrogate data. Shaded areas indicate *SEM*. (b) Same as (a), for sensitivity

Next, we looked at the spectral data separately for trials with a motor response that was spatially congruent versus incongruent with the one in the previous trial (Figure 5).

While the overall spectral pattern was similar to the grand‐average, we observed no significant peaks, neither in the RT nor in the sensitivity data (all *p*s > .417).

**FIGURE 6:**
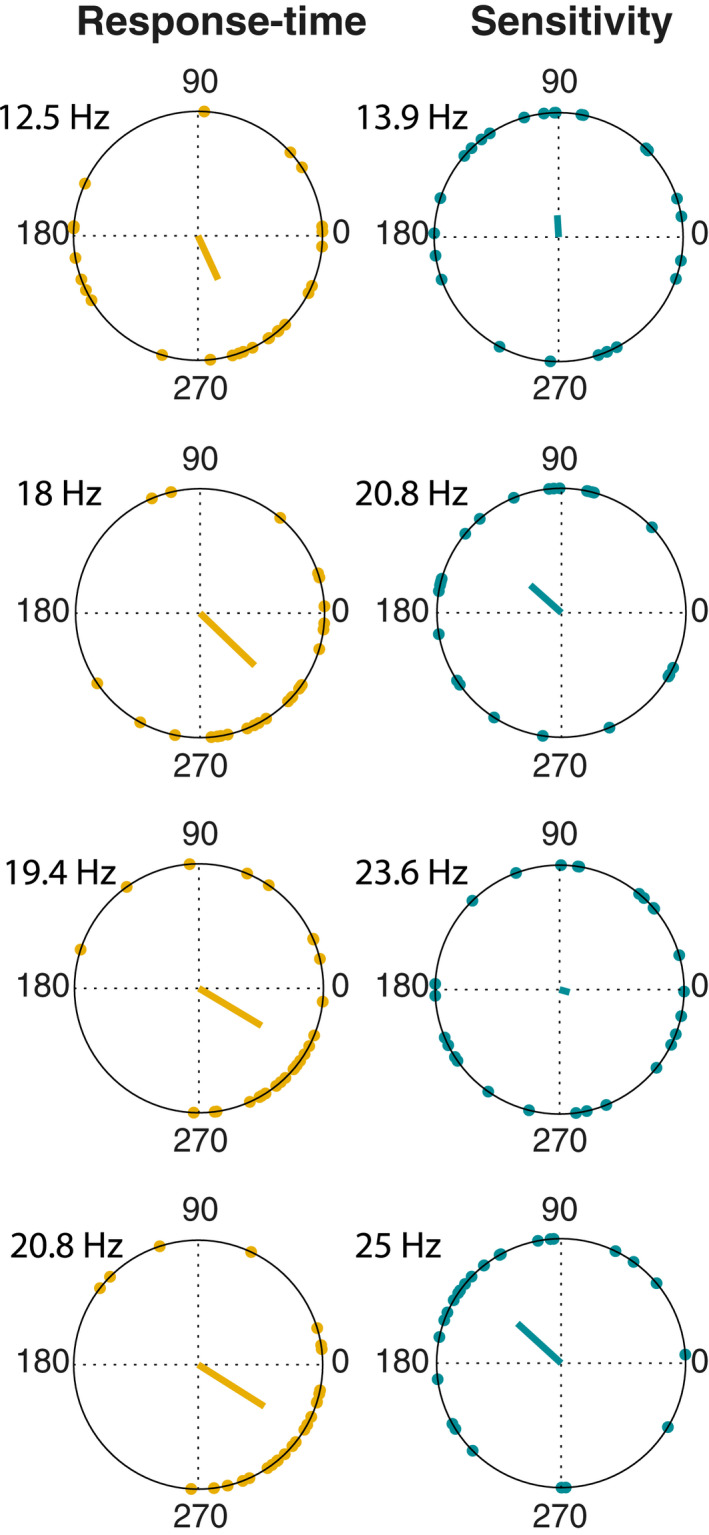
Phase angles for significant fluctuations. Phase angles of response‐time (left column) and sensitivity (right column) fluctuations for all significant peaks in the spectrum. Dots indicate individual participants, vectors represent the mean phase across participants

Finally, we investigated the phase consistency across participants for all significant spectral peaks (Figure 6). For the grand‐average RT time course, the Rayleigh test yielded significant results for all observed spectral peaks, that is, at 12.5 Hz (*p* = .015), 18.1 Hz (*p* < .001), 19.4 Hz (*p* < .001), and 20.8 (*p* < .001), indicative of a non‐uniform distribution of phase angles around the circle. For the sensitivity time course, we observed significant results for the peaks at 20.8 Hz (*p* = .049) and 25 Hz (*p* < .001).

Note that to investigate a potential impact of the visual target on subsequent behavioral oscillations, we additionally analyzed our data time‐locked to the current target (rather than the preceding button press). This analysis did not yield any significant spectral peaks (see Figure S3).

## 4 DISCUSSION

Our study set out to investigate cyclic fluctuations in the capacity for response initiation as potential origin of the commonly observed intraindividual RT variability. While we observed such fluctuations in RTs in the alpha/mu and beta frequency range (12–20 Hz) relative to the most recent motor output as a reset signal, the concurrently present fluctuations in sensitivity suggest the underlying presence of additional perceptual‐ or attentional mechanisms.

### 4.1 Motor action induces 12–20 Hz oscillations in response times

In line with our expectations, we found significant peaks in the spectral energy of RT time‐courses, relative to the preceding button press. In other words, the speed of a given response fluctuated cyclically as a function of the time since the last response. With a frequency of 12–20 Hz, these fluctuations cover the range of sensorimotor rhythms (alpha/mu and beta), whose involvement in the preparation and execution of motor output is well known (Desideri et al., 2019; Hussain et al., 2019; Kilavik et al., 2013; McFarland et al., 2000; Pogosyan et al., 2009; Stolk et al., 2019; Tzagarakis et al., 2010). This could indicate that our RT finding reflects patterns of waxing and waning neural excitability in cortical motor areas, instantiated by sensorimotor rhythms. As such, our results would be in line with the aforementioned TMS studies, which reported modulations in motor‐evoked potentials dependent on the pre‐stimulation sensorimotor oscillatory phase (Berger et al., 2014; Keil et al., 2014; Khademi et al., 2018), by demonstrating this phase can be reset by a preceding motor output.

Importantly, unlike virtually all previous dense‐sampling studies (e.g., Benedetto & Morrone, 2017; Diederich et al., 2012; Drewes & VanRullen, 2011; Helfrich et al., 2018; Peters et al., 2021), our experiment employed a decidedly simple discrimination task using highly salient stimuli. Arguably, this avoids that results depend on more complex task representations and brings the situation much closer to the most basic motor‐response situations, in which humans quickly and accurately respond to sudden, salient and potentially threatening external events. The fact that cyclic fluctuations in performance are nevertheless readily observable under such circumstances, thus, provides important novel support for the generality of the phenomenon. Moreover, while both fluctuations in RTs relative to an external sensory stimulus (Benedetto & Morrone, 2017; Diederich et al., 2012; Drewes & VanRullen, 2011; Helfrich et al., 2018) and fluctuations in hit rates relative to a motor response (Benedetto et al., 2016, 2018; Hogendoorn, 2016; Nakayama, 2019; Tomassini et al., 2017; Zhang et al., 2019) have previously been reported in separate experiments, our present study links these phenomena and demonstrates oscillations in RTs reset by the preceding button press (cf. Bellet et al., 2017). However, we note that our data do not discriminate whether these oscillations reflect motor processing in the narrowest sense (e.g., activity in pyramidal cells in M1; cf. Lacey et al., 2014), to the sensory feedback provided by motor execution (cf. Tan et al., 2016), or to the preparation of a motor response, that is, to motor processing in a broader sense.

### 4.2 Concurrent (14–25 Hz) oscillations in sensitivity hint at sensory contribution

Commonly, variability of behavioral outcomes in 2‐AFC tasks are accredited to noise during (1) sensory processing, (2) decision‐making, and (3) motor response generation (Dmochowski & Norcia, 2015; Hanes & Schall, 1996; Luce, 2008; MacDonald et al., 2006; Wood, 1977). Out of those three, noise in (1) and (2) can affect both RTs and sensitivity, while noise in (3) should only affect RTs (see, e.g., Huang et al., 2015; Lingnau & Vorberg, 2005; Sumner & Brandwood, 2008; for effects of motor priming). Thus, by itself, our result of significant RT fluctuations could be considered as evidence for cyclic fluctuations in motor excitability, independent of perceptual‐ or attentional mechanisms, especially considering the presently used simple task and salient stimuli. However, on the one hand, we concurrently observed significant spectral peaks in the time‐course of sensitivity, in frequency bands largely overlapping with those found in RT results (14–25 Hz). On the other hand, they could have an origin in perceptual processing, and might more readily be explained by oscillations of excitability in sensory areas. Indeed, similar fluctuations in sensitivity or hit rates, albeit lower in frequency (~4–10 Hz), have been reported in previous studies using more difficult tasks titrated to be close to perceptual threshold, and were commonly attributed to perceptual or attentional mechanisms (Fiebelkorn et al., 2013; Helfrich et al., 2018; Landau & Fries, 2012; Re et al., 2019). Therefore, it is possible that ongoing fluctuations in the ability to process incoming sensory input may lead to sensory variations in both, whether or not a stimulus is categorized accurately and how long that categorization takes.

However, the fact that we did not observe significant performance fluctuations time‐locked to the visual target stimulus (see Figure S2) indicates that trial‐by‐trial variations in the sensory noise were small, presumably due to the highly salient character of the stimulus. Moreover, our data do not show characteristics of a speed‐accuracy trade‐off: Participants were both faster and more accurate in incongruent compared to congruent trials, and the time series fluctuations for RTs and sensitivity at the significant spectral frequencies did not exhibit a consistent phase relationship (i.e., at any given moment, RTs do not correlate either positively or negatively with sensitivity). This suggests, that our RT and sensitivity fluctuations did not arise within the same processing stage (i.e., either at the sensory or decisional level), but more likely at different stages, likely involving motor areas.

Finally, the lack of a priming effect (incongruent trials being faster and more accurate than congruent ones) may indicate that our task is not optimal to investigate sensory processes linked with perceptual sensitivity but results more likely reflected motor or decisional processes. Indeed, there is evidence that biases rather than sensitivity oscillates in the alpha–beta range (e.g., Benedetto et al., 2019; Ho et al., 2017; Zhang et al., 2019).

A recent series of studies has started to shed light on the possible origins of such fluctuations in behavioral performance linked to motor actions (Benedetto et al., 2016, 2021; Benedetto & Morrone, 2017; Tomassini et al., 2015, 2017; see Benedetto et al., 2019, for a review). For instance, using electroencephalography (EEG), Tomassini et al. (2017) demonstrated that theta oscillations in visual cortex are both aligned to the initiation of an upcoming hand movement up to 1500 ms later and predictive of task performance in a visual task unrelated to the movement. Along with other studies (Bellet et al., 2017; Hogendoorn, 2016; Wutz et al., 2016), this finding indicates that already the preparation of a motor output, even if task‐irrelevant, can modulate the phase of ongoing neural oscillations in sensory areas, presumably via corollary discharge signals (Benedetto et al., 2019; Rolfs et al., 2013; Schroeder et al., 2010; Tomassini et al., 2017). Alternatively, the causality might be reversed, and a sensory rhythm might define cyclic windows of facilitated action preparation, in synchrony with attentional sampling (Benedetto et al., 2019; Nakayama & Motoyoshi, 2019). Regardless of the actual neural implementation, such a tight temporal coordination between motor and sensory processing is an integral prerequisite for the action‐perception loops proposed in models of action control (Hommel, 2004; Wolpert et al., 1995) and embodied cognition(Benedetto et al., 2019; Gibson, 1962; Melloni et al., 2009; Schroeder et al., 2010), and is necessary for the joint representation of stimuli and motor responses in task representations (e.g., Frings et al., 2020; Hommel, 2004).

In summary, while the present data are well in line with these previous studies and theoretical considerations, one limitation of our design is, that despite the simple task and highly salient stimuli, response initiation is still depended on preceding perceptual and attentional processes, making it difficult to unambiguously disentangle the contribution of motor‐ versus sensory processes.

### 4.3 No evidence for alternate prioritization of left‐ and right‐hand responses

One aim of our study was to investigate whether RT fluctuations in congruent and incongruent trials might be in counterphase, indicating an alternating prioritization of left‐ and right‐hand responses over time. Such a mechanism is conceivable, given the previously demonstrated alternating prioritization of attended locations (Landau & Fries, 2012), objects (Fiebelkorn et al., 2013), or memory templates (Pomper & Ansorge, 2021).

While we observed significant fluctuations of behavioral performance pooled across all trials, separate analyses of congruent and incongruent trials revealed no significant peaks, thus, prohibiting any subsequent investigation of their phase relationship. A likely reason for this null finding might be that splitting the data into congruent and incongruent responses resulted in an insufficient signal‐to‐noise ratio for the detection of behavioral oscillations (Fiebelkorn, 2021). As an alternating prioritization between different motor outputs on sensorimotor rhythmic temporal scale is indicated by previous electrophysiological work (Pfurtscheller et al., 2006; Takamatsu et al., 2021), future behavioral dense‐sampling studies using higher trial numbers might successfully uncover this phenomenon.

On a trial‐by trial level, however, our data show pronounced sequential effects, with incongruent responses being both faster and more accurate than congruent responses.

Due to the constraint of not presenting more than four trials requiring the same response in succession, eventually incongruent trials were more likely than congruent trials. Thus, this result suggests that participants established a bias for response alternation on a slower temporal scale, indicative of their higher subjective expectancies for response switches or “incongruent trials” in the current article's terminology (Bertelson, 1961; Cho et al., 2002; Ho et al., 2019).

### 4.4 Speed of performance fluctuations likely reflects task characteristics

With frequencies from 12–25 Hz, our observed fluctuations are about twice as fast as many of those reported in the past, which commonly found spectral peaks at 4–10 Hz (Benedetto et al., 2019; Fiebelkorn et al., 2013; Fiebelkorn & Kastner, 2019; Landau & Fries, 2012; Pomper & Ansorge, 2021; VanRullen, 2016).

One obvious possibility would be the involvement of faster sensorimotor mu and beta neural oscillations in our study, given that the relevant reset event is a motor output rather than an external stimulus as used in most previous works(but see Bellet et al., 2017). However, several other studies nevertheless reported slower behavioral oscillations in the theta and lower alpha (4–7 Hz and 8–12 Hz, respectively) range, despite also analyzing the data relative to a preceding motor output (Benedetto et al., 2016, 2018; Hogendoorn, 2016; Nakayama & Motoyoshi, 2019; Tomassini et al., 2017; Zhang et al., 2019).

Alternatively, the increase in the speed of performance fluctuations could be related to our decidedly simple task design and salient target stimulus. More difficult tasks likely require more complex and thorough stimulus processing, potentially resulting in lower attentional sampling rates and increased motor response criteria associated with slower fluctuations in motor excitability. Indeed, studies have demonstrated proportionate reductions in the speed of performance fluctuations related to increasing task difficulty (Chen et al., 2017; Holcombe & Chen, 2013; Re et al., 2019).

Notably, our data do feature peaks at a lower frequency range (<10 Hz, particularly at 5.6 Hz for RT data and at 2.8 Hz for sensitivity data), albeit not statistically significant. Out of those, the peak at 2.8 Hz is lower than most previous observations of behavioral oscillations and outside of the presently hypothesized relevant frequencies, particularly in the alpha and beta, but also the theta range. However, it is possible that a subset of participants exhibited rhythmic fluctuations in RTs in the lower frequency range, at around 6 Hz (see also Figure S2), in line with either a sub‐harmonic of the significant alpha peak at 12.5 Hz, or an additional independent oscillatory process at a frequency closer to most recent reports of behavioral rhythms (e.g., Fiebelkorn et al., 2013; Landau & Fries, 2012; Pomper & Ansorge, 2021).

Finally, it is worth mentioning that a recent evaluation of several dense‐sampling experiments has particularly questioned the relevance of reported fluctuations in the lower frequency range, which might artefactually result from spectral decomposition of non‐oscillatory components in the behavioral performance time‐course (Brookshire, 2021). According to this account, the presently observed higher frequency peaks (> 12 Hz) are more likely to be of physiological than artefactual origin, compared to previously reported lower frequency fluctuations.

## 5 CONCLUSIONS

Our present study reports cyclic fluctuations in RTs and sensitivity relative to a preceding motor action. Importantly, unlike previous dense sampling experiments, we used a simple task combined with highly salient stimuli and demonstrate that the phenomenon generalizes to less complex, cognitively less demanding protocols closer to everyday life. Moreover, our work further adds to the literature by demonstrating behavioral fluctuations at a relatively high speed, which might be a consequence of both low task difficulty and the involvement of sensorimotor rhythms.

While the observed RT fluctuations of 12–20 Hz fit well with neural mu and beta oscillations reflecting cyclic changes in motor excitability, our concurrently observed peaks in the spectrum of the response sensitivity time‐course suggest at least a partial dependence on perceptual or attentional mechanisms.

## AUTHOR CONTRIBUTIONS

**Ulrich Pomper:** Conceptualization; data curation; formal analysis; funding acquisition; investigation; methodology; software; visualization; writing – original draft. **Ulrich Ansorge:** Conceptualization; funding acquisition; project administration; resources; supervision; writing – review and editing.

## CONFLICT OF INTEREST

The authors declare no competing financial interests.

## Supporting information


**Appendix S1** Supporting Information

## Data Availability

Data are available from the corresponding author upon reasonable request.
