# Supplementary material for: Motor‐induced oscillations in choice response performance
Source: Psychophysiology. 2022 Aug 30;60(2):e14172. doi: 10.1111/psyp.14172 (PMC10078311; doi:10.1111/psyp.14172)
Supplement: Supplementary file 1 — Appendix S1 Supporting Information [file PSYP-60-0-s001.docx]

Supplementary Material


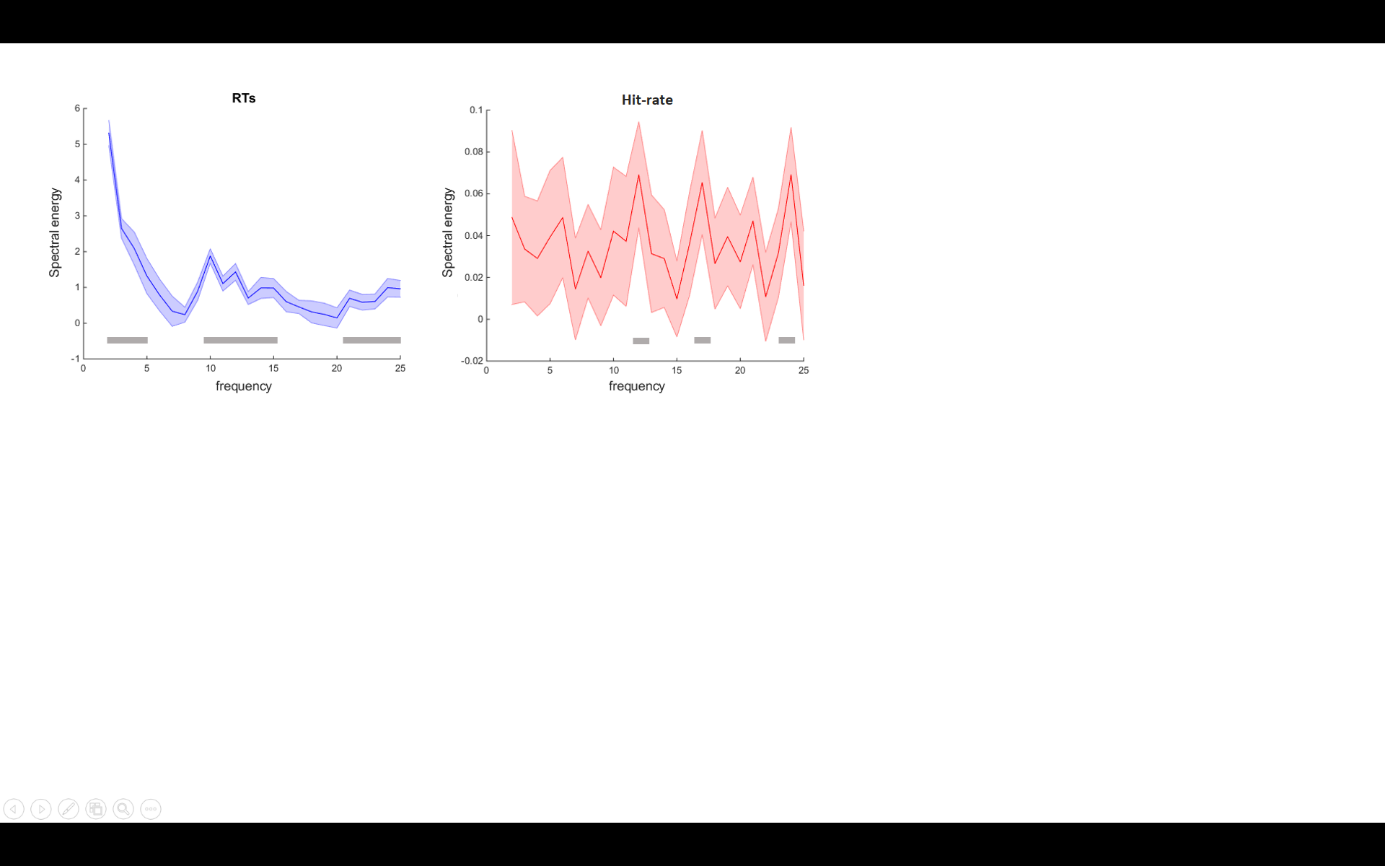


*Figure S1: Spectral results computed via fitting, for each frequency, a logistic regression with a sine and a cosine function as predictors.*

*In addition to the spectral analysis via FFT and subsequent permutation statistics, we have performed a logistic regression analysis (as described in Tomassini et al., 2017). For each participant’s RT and accuracy data, we fitted logistic regression models including as predictors a sine and a cosine of a given frequency in the range from 2 to 25 Hz (in steps of 1 Hz). Note that, since this procedure uses raw, un-binned single trial data as input, we used accuracy instead of sensitivity (d’), as the latter requires binned data from several trials for the calculation of hit- and false alarm rates. We then performed a group-level (random effects) analysis by testing the average of the individual participants’ beta coefficients b1 and b2 against zero via a bivariate Hotelling’s T-square statistic. The standard error of the beta coefficients was calculated via a jackknife procedure.*

*For RTs, we found significant spectral peaks at 2 to 5 Hz (all ps < .002), 9 to 15 Hz (all ps < .004), and 21 to 25 Hz (all ps < .05). For accuracy, we found significant spectral peaks at 12 Hz (p < .04), 17 Hz (p < .03), and 24 Hz (p < .02). Overall, this alternative analysis procedure corroborates our main results obtained by a combination of FFT spectral analysis and permutation statistics.*

*Left: Grand average spectral representation of response time (RT) courses. Right: Grand average spectral representation of hit-rate time courses. Grey bars indicate significant spectral peaks at the group level (p < 0.05). Shaded areas indicate the jackknife standard error.*


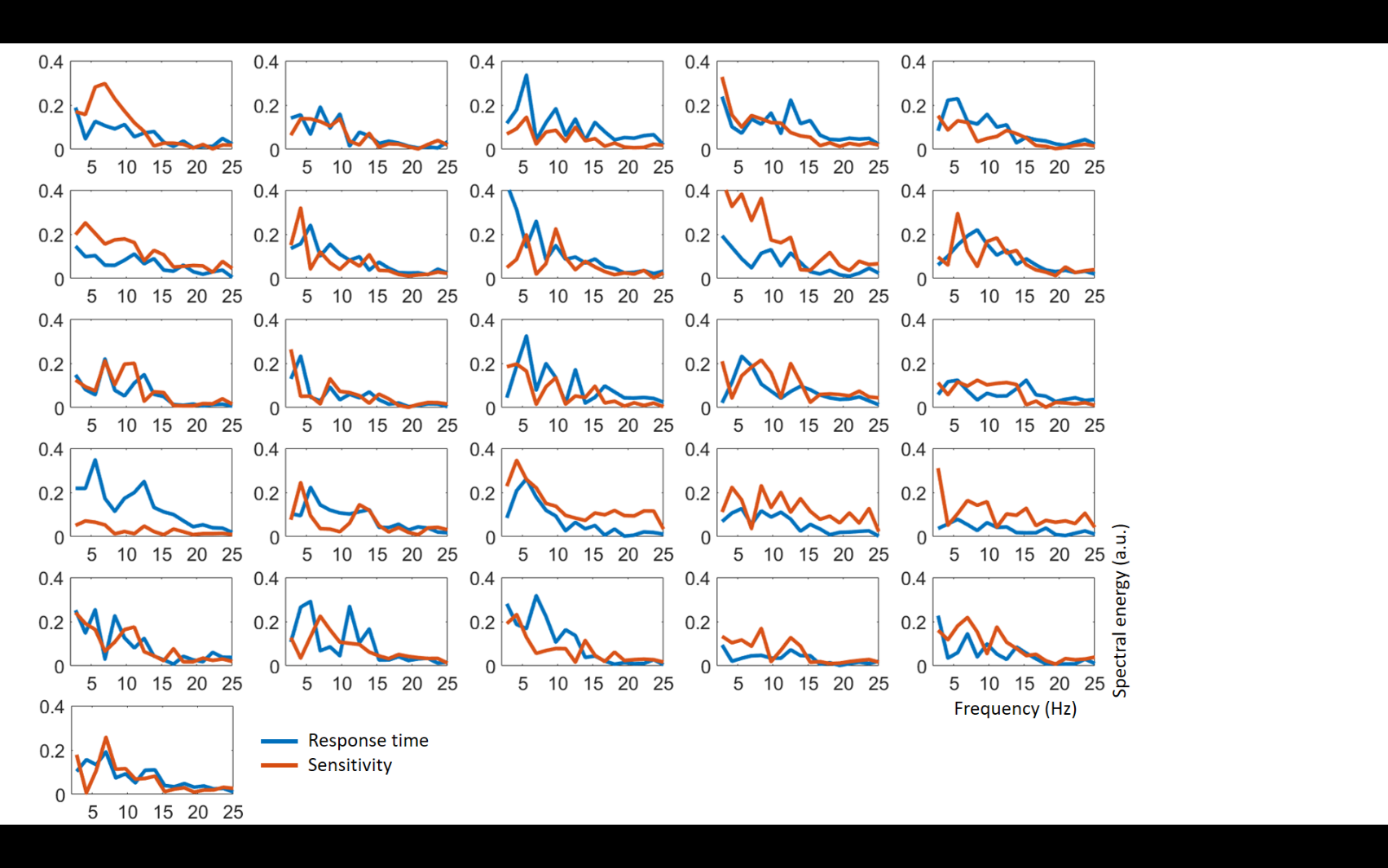


*Figure S2: Spectral representation of performance time courses for individual participants. Spectra for response times are depicted in blue, spectra for sensitivity (d’) in red. Peaks in the low beta frequency range (12–25 Hz) are present across all participants, although expectedly, the exact peak frequency varies.*


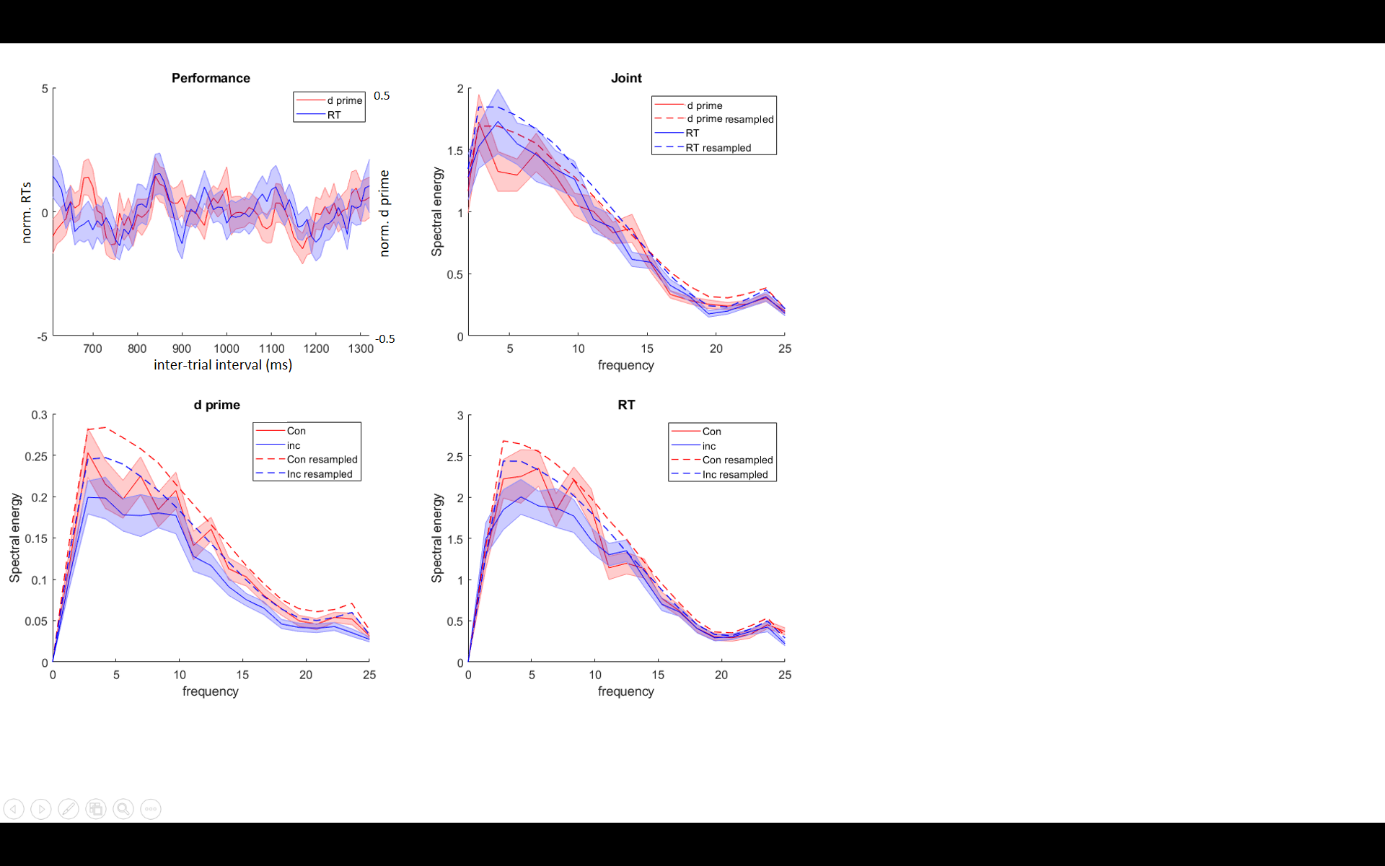


*Figure S3: Stimulus-locked analysis. Upper left: Average response times (RTs, blue trace) and sensitivity (d’, red trace) as a function of the variable inter-trial interval (ITI), pooled across all trials. Upper right: Grand-average spectral representation of performance time courses. Blue traces indicate the power spectrum for fluctuations in RTs, red traces for fluctuations in sensitivity. Solid lines represent the empirical data, dashed lines the threshold determined from resampled surrogate data. Lower left: Spectral representation of sensitivity time-courses separately for congruent and incongruent trials. Red traces indicate the power spectrum for fluctuations in*

*congruent trials, blue traces for fluctuations in incongruent trials. Solid lines represent the empirical*

*data, dashed lines the resampled surrogate data. Lower right: Same as lower left, for RTs. Shaded areas indicate SEM. norm.: normalized; Con: congruent; Inc: incongruent*
